# Reviewing the Impact of Maternal Opioid Use Disorder on Fetal Development and Long-Term Pediatric Health Outcomes

**DOI:** 10.7759/cureus.72192

**Published:** 2024-10-23

**Authors:** Manahil Asad, Felicia T Bonner-Reid, Fawaz Aldoohan, Laura M Marrelli, Neisha Ghanie, Hussein Attia Hussein Mahmoud, Sruthi Venkatraj Srividya, Preanka Devadas Gandhi, Muneeza Zehra, Zahra Nazir

**Affiliations:** 1 Medicine and Surgery, Fauji Foundation Hospital-Foundation University Medical College (FUMC) Islamabad, Islamabad, PAK; 2 Medicine and Surgery, University of Medical Sciences of Manzanillo (Celia Sánchez Manduley), Manzanillo, CUB; 3 Family Medicine, American Academy of Research and Academics, Newark, USA; 4 Psychiatry, Sapienza University of Rome, Rome, ITA; 5 College of Medicine, American University of Antigua, Coolidge, ATG; 6 Diagnostic Radiology, Heliopolis Hospital, Cairo, EGY; 7 Medicine and Surgery, University of Central Lancashire, Preston, GBR; 8 Graduate Medical Education, Metropolitan University College of Medicine, St. John's, ATG; 9 Internal Medicine, Karachi Medical and Dental College, Karachi, PAK; 10 Internal Medicine, Combined Military Hospital (CMH) Quetta, Quetta, PAK

**Keywords:** methadone, naltrexone, neonatal care, opiod abuse, pregnancy

## Abstract

Opioid use during pregnancy has emerged as a notable public health concern with far-reaching impacts on both maternal and child health. This review investigates the consequences of opioid use disorder (OUD) and maternal opioid use during pregnancy, focusing on fetal and pediatric outcomes and available treatment options. The study focuses on the rising prevalence of opioid use among pregnant women, with emphasis on the adverse effects on neonates, including neonatal abstinence syndrome (NAS), preterm birth, low birth weight, and long-term cognitive and behavioral deficits. Furthermore, it explores the neurological implications of opioid exposure on fetal brain development, emphasizing disrupted synaptic plasticity, oligodendrocyte differentiation, and myelination. Current treatment strategies such as opioid maintenance therapy (OMT) with methadone and buprenorphine are discussed, including their benefits in improving prenatal care adherence but also their associated risks, such as NAS and small birth weights. Maternal OUD underscores the need for a multidisciplinary approach to manage opioid dependence in pregnant women effectively and calls for more comprehensive research to address the long-term developmental impacts on children, as well as to explore more effective prevention and intervention strategies for maternal OUD.

## Introduction and background

The umbrella term “opioid” refers to the extracted alkaloids from the natural poppy seed, namely, morphine and codeine, inclusive of their synthetic and semi-synthetic derivatives, each with unique properties and adverse effects [1].

Chronic dysregulation of the dopaminergic mesolimbic reward system in the brain is the pathophysiology of addiction to opioids and other illicit substances [2]. The primary target of opioids, either exo-or endogenous, exerts their function through the interaction of the mu-receptor, a member of the superfamily of seven transmembrane receptors. This interaction produces both the analgesic effect and addictive properties [3]. Mu receptors are expressed on neurons in many brains, particularly on the rostral ventromedial medulla, and send descending projections to the spinal cord. These receptor agonists also modulate spinal nociception, which may contribute to opioid hyperalgesia effects [4]. The mesolimbic dopaminergic system, basolateral amygdala, extended amygdala, and brain and hormonal stress systems are the sites that are mainly altered to give the withdrawal symptoms in contrast to the peripheral opioid receptors, the locus coeruleus, and the periaqueductal gray matter that give rise to the somatic signs of withdrawal [5].

Opioid use constitutes the most significant portion of the global burden of disease, as it encroaches on every aspect of societal life [6]. This synthetic or semi-synthetic compound has the advantage of reducing pain and causing sedation at the expense of dependability due to positive reinforcement [6]. Recent data by AH Hirai et al. showed that from 2010 to 2017, maternal opioid use increased by more than 100% in the US across different demographics [7]. Notably, an alarming 333% increase in the prevalence of OUD was noted in the analyzed data from the national and state inpatient sample databases encompassing the period 1999 to 2014, with a significant rate increase from 1.5 to 6.5 cases per 1,000 deliveries [8]. Data from Truven Health’s MarketScan Commercial Claims and Encounters and Medicaid depicted that outpatient opioid was prescribed to 39.4% of women who were Medicaid-enrolled, compared with 28% of women who had private insurance [9]. Low birthweight and preterm band birth can occur as a result of opioid use, and to combat this, the World Health Organization recommended guidelines institute opioid maintenance therapy (OMT) with methadone and buprenorphine during pregnancy to increase medication compliance and follow-up of prenatal care [10]. However, this also comes with its side effects, with methadone being associated with small birth weight and neonatal opioid withdrawal syndrome (NOWS) [10].

Opioid exposure in the prenatal period has its share of risks, including relative microcephaly and deficits in motor, language, and cognitive domains [11]. However, the risks continue even after this period. Lee SJ et al. did a systematic review and meta-analysis, across 16 studies comparing exposed and non-exposed children or infants to opioids, which showed lower infant cognitive scores, psychomotor scores, and lower IQ with standardized mean differences of 0.77, 0.52, and 0.76, respectively [12].

This review paper aims to investigate maternal opioid use or dependence during pregnancy, to elucidate its impact on fetal and pediatric outcomes, and to discuss the treatment options, strategies, and policies available by reviewing recent literature. 

## Review

Prevalence and challenges of maternal opioid use

The pain women experience during childbirth is immense. It is affected by a multitude of factors with varying intensity, pharmacological interventions that aid in pain management, and pharmacological interventions (mainly opioids) that provide pain relief. While the former might be safer for the mother and the baby, its efficacy is unknown. By contrast, despite having more evidence to support its efficacy, the latter has more associated adverse effects [13]. 

Substance use disorder (SUD) among pregnant women is a significant public health issue. Both prescription opioid use and illicit opioid abuse have severely increased in recent years. Opioids act at the receptors in the central and peripheral nervous system, blocking the pain intensity of pain signals and leading to euphoria and analgesic effects. Prolonged use can result in both physical tolerance and dependency [14]. Opioid use disorder (OUD) is characterized by craving, tolerance, and continued use of opioids despite adverse consequences. It is managed successfully by combining medications with support and behavioral therapy [15].

 Maternal OUD has significantly escalated over the years, with an increase from 1.19 to 5.63 per 1,000 hospital births from 2000 to 2009 [16]. However, since both studies were conducted in the United States, the demographic may vary accordingly in different countries. On the contrary, 2023 research conducted in Maine showed a significant decrease in that region from 35.3 to 18.8 per 1,000 deliveries from 2016 to 2022 due to many reasons, which included better prevention of opioid use and misuse among girls and women [17]. 

Opioid use during pregnancy could result in maternal, fetal, and infant complications and an overdose leading to death in postpartum [18]. In fetuses and infants, the complications include neural tube defects, congenital heart defects, and gastroschisis [19]. As of 2017, approximately 5.8 in 1000 pregnancies are complicated using opioids. However, since this is based in the United States, results may vary widely by region [20]. The increase in maternal OUD has led to the escalation of neonatal abstinence syndrome (NAS), a postnatal drug withdrawal disorder affecting newborns [20]. NAS is a multi-system disorder with many neurological and gastrointestinal symptoms. Prenatal opioid exposure in children is also known to stunt growth rate and development while diminishing their cognitive ability [21].

Averting such complications requires instituting a universal screening program during the gestational period to mitigate them while also providing the requisite treatment programs to assist pregnant women [18].

Factors contributing to opioid use during pregnancy

The prevalence of co-occurring substance use and comorbid mental illness exacerbates the adverse effects of opioid use. Tobacco smoking is as high as 95%. Up to 35% of pregnant women with OUD also use cocaine, cannabis, and benzodiazepines concurrently. A history of trauma and comorbid psychiatric diagnoses, such as depression, anxiety, bipolar disorder, PTSD, and personality disorders, are also significantly more common in pregnant women with OUD. Poor nutrition, prenatal care, poverty, long-term medical issues, and domestic abuse are also more common. The detrimental consequences of prenatal opioid exposure may be exacerbated by untreated OUD, which can also result in early dysfunctional mother-infant relationships and interrupted parental care [22]. It is recommended that pregnant and postpartum women take medication for maternal opioid use disorder (MOUD), such as methadone or buprenorphine [23]. However, patients on methadone and buprenorphine are at risk of relapsing, abusing heroin or other drugs throughout their pregnancy, participating in activities that could compromise their prenatal care, or having other mental health issues that could complicate the risk assessments [23]. A longitudinal, retrospective study using a provincial-level linkage of seven health administrative databases between April 1, 2000, and March 31, 2019, in British Columbia, shows that the annual incidence of women with OUD in pregnancy has increased over threefold, from 166 cases identified in 2000-2001 to 513 in 2018-2019 [23]. It assesses tobacco smoking, alcohol consumption, or any prescription, nonprescription, or illicit opioid use (as determined by the prenatal care provider to be a risk to the fetus), as well as other nonopioid and nonalcohol substance use or tobacco dependency during pregnancy.

Related comorbid conditions are extensive and comprise the following: alcohol use disorder, nonopioid and nonalcohol SUD (92%), infectious diseases (HIV, hepatitis C virus), mental health conditions (92%; anxiety, depression, bipolar disorder, schizophrenia, and other psychoses, stress and adjustment disorder, personality disorders, attention-deficit/hyperactivity disorder, and developmental disorder), chronic pain (69%), and pain medications received during labor [23]. Gestational diabetes reported polysubstance use during pregnancy, and urban county of residence was another factor that was significantly linked to medication for opioid use receipt. All of these factors increase the odds of receiving medication for OUD during pregnancy and after delivery [24]. Abdominal pain, arthritis or arthropathies, and back and neck pain were the most frequently identified pain problems across all opioid users. Migraines were also common. Although patients' pain treatment needs vary greatly, information on opioid-specific outcomes can help prescribers choose an opioid to treat pain in the late stages of pregnancy [25]. The rising trend of opioid use is causing more significant complications, especially among pregnant women, like NOWS, or hindering the neurological development of newborns. It has become ever more important to diagnose opioid use in pregnant women as early as possible, and awareness of the complications should be raised in order to initiate pharmacological or nonpharmacological therapy, which has been shown to reduce both maternal and neonatal complications of opioid use and aid in the recovery of this disorder.

Related comorbid conditions diagnosed prior to delivery are extensive and comprise the following: alcohol use disorder, nonopioid and nonalcohol SUD, infectious diseases, mental health conditions, chronic pain, and pain medications received during labor [23].

Effect of opioids on fetal development, including the pathophysiology of opioids affecting the fetus

Opioids cause significant changes to the hippocampal region, most likely due to their effect on the regulation of hippocampal development [26-28]. The hippocampus, an area of the brain important to learning and memory, is known to undergo physiological events related to the formation of synaptic connections and plasticity that are usually inhibited by opioids. Because of these opioids' impacts on the signaling pathways necessary to maintain the neural map's flexibility (which may promote cognitive development), there is a high vulnerability to these effects. Research has shown that the simultaneous release of opioids and glutamate at the neuronal extrasynaptic level is involved in initiating long-term potentiation (LTP), which is responsible for strengthening synapses and memory. The co-release is crucial for LTP induction at the synapses formed by Schaffer collaterals, where the lateral perforant pathway (PP) connects to dentate gyrus (DG) granule cells and CA3 pyramidal neurons; it is thus necessary at the synaptic locations where CA3 pyramidal neurons are the target of mossy fiber projections from DG granule cells. The interactions between neurotransmitter systems that control the mechanisms of synaptic plasticity (which, again, are essential to memory processes along with learning in the hippocampus) are underlined by the combined action of opioids and glutamate at these synaptic connections [26-28].

**Figure 1:**
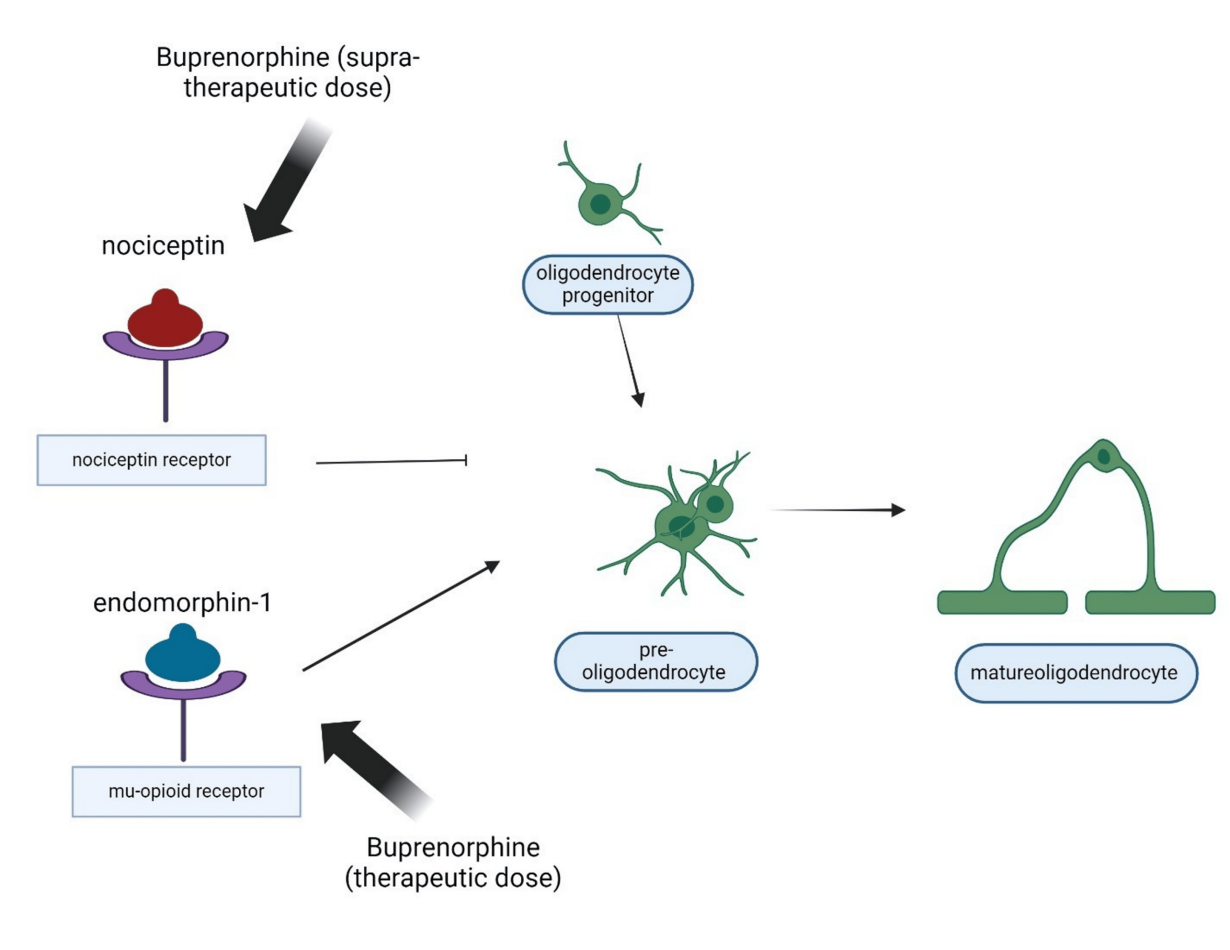
Effects of buprenorphine on oligodendrocyte maturation via Mu-opioid and nociception receptor pathways Source reference: [30] Created by Laura M. Marrelli using biorender.com

Some modulatory effects of opioids on perinatal brain development, specifically affecting oligodendrocyte differentiation and myelination, are highlighted in a recent study by Velasco et al. [29]. This study, along with others, is crucial to our understanding of the effects of opioids on fetal development. Research employing both animal models and cell cultures appears to indicate that the control of the timing of oligodendrocyte development and myelinating processes depends on the balance between mu-opioid and nociceptin receptor activation. Exogenous opioids (such as methadone and buprenorphine) may disrupt this balance, leading to potential abnormal brain myelination and impaired axonal connectivity. Imaging studies in infants exposed to opioids prenatally have, as a matter of fact, shown white matter injuries and altered myelin structures. In addition, emerging evidence indicates that opioid exposure can induce significant transcriptional changes in oligodendrocytes, with potential impacts on brain maturation, especially in populations like adolescents and young adults, who are undergoing critical periods of late-stage myelination. This could alter the normal maturation process of the developing brain [30]. Figure 1 depicts the effects of buprenorphine on oligodendrocyte maturation via different pathways.

According to Griffin et al. [31], norbuprenorphine, which is a primary metabolite of buprenorphine, is known to have the potential to cause prenatal opioid dependency and, thus, NOWS. Neonatal receptors and endogenous peptides have unique properties, explaining why the presence of opioid receptors in developing fetuses may have different effects than exposure in adults. Opioid receptors are found throughout the central nervous system and peripheral tissues, including the uterus and placenta [32,33]. Studies found that the endogenous opioid system is highly involved in neurogenesis and the control of oligodendrocyte myelination; hence, disruption of normal brain maturation by exogenous opioids could be considered a possibility [30]. Moreover, opioids raise dopamine levels in the brain, which might impact dopamine-rich regions such as the basal ganglia, frontal cortex, and nucleus accumbens, leading to neurocognitive deficiencies [34,35]. Moreover, oxidative stress, decreased cell proliferation, and increased apoptosis in crucial brain areas, such as the striatum and hippocampus, are some of the ways by which opioids may cause neurological damage [36-38]. However, there might be some biases due to the fact that it is difficult to distinguish between direct and indirect effects on the fetus since the physiological effects of opioids on the mother, such as changed production of stress hormones, can create a significant confounder.

Moreover, glutamatergic signaling appears to be involved in a significant way in the psychopathology of prenatal opioid exposure. Different studies have found altered glutamate receptor expression and disrupted glutamatergic transmission, primarily within the hippocampus, in animal models exposed to opioids before birth [39,40-42]. On the other side, the simultaneous co-administration of dextromethorphan, an NMDA receptor antagonist, during fetal methadone exposure prevented an increased preference for methadone in adult rats, putting forward the claim that there could be a protective effect against opioid-induced behavioral changes [43]. These findings hint at a drug-induced imbalance in glutamate signaling within the striatum, which might have an impact on the neuropathophysiology associated with opioid abuse [44-46].

Kang et al. [47] suggest that prenatal opioid exposure during the first trimester, at higher doses, and for prolonged periods can cause an increased risk of psychiatric disorders in children. It is noted that such exposure modestly raises the risk of severe neuropsychiatric conditions, mood disorders, intellectual disabilities, and attention-deficit hyperactivity disorder (ADHD). The study highlights again that excessive opioid use, particularly during early pregnancy and in the context of cesarean sections, may require caution due to potential links with brain developmental disorders in offspring. Table 1 table outlines how opioid exposure affects brain development, impacting synaptic plasticity, neurogenesis, and neurotransmitter signaling and increasing the risk of cognitive and psychiatric disorders, especially in prenatal exposure.

**Table 1:** Opioid exposure affecting brain development, impacting synaptic plasticity, neurogenesis, and neurotransmitter signaling

Key findings	Mechanisms and effects
Opioids affect hippocampal plasticity and synaptic connections, leading to cognitive development issues.	Inhibition of synaptic plasticity, reduced neural connectivity in the hippocampus [26-27]
Opioids influence oligodendrocyte differentiation and myelination, potentially causing abnormal brain development.	Disruption of oligodendrocyte maturation, white matter injuries [29-30]
Prenatal opioid exposure can lead to Neonatal Opioid Withdrawal Syndrome (NOWS) and disrupt neurogenesis.	Dependence and withdrawal symptoms, interference with neurogenesis [30-31]
Altered glutamatergic signaling associated with prenatal opioid exposure may contribute to neuropsychiatric disorders.	Altered glutamate receptor expression, disrupted transmission [40-42]
Increased dopamine levels due to opioids may cause neurocognitive deficits in brain regions rich in dopamine.	Impact on nucleus accumbens, basal ganglia, and frontal cortex [34-35]
Opioid exposure during early pregnancy is linked to a higher risk of psychiatric disorders in children.	Higher risks of ADHD, mood disorders, and intellectual disabilities [47,48,49]

Immediate and long-term risk of the fetus with an opioid-using mother, including delayed developmental milestones

Fetal opioid exposure is linked to several adverse growth outcomes for fetuses; indeed, infants exposed to opioids during pregnancy are often born with lower average birth weights than those not exposed [50-54]. Moreover, premature infants seem to have had higher total opioid exposure during pregnancy [55,56].

Most infants that were exposed to opioids surprisingly score within the normal range on early neurodevelopmental tests [52,54] but often perform worse than non-exposed peers in motor, cognitive, and behavioral assessments during their first year of life [53,57]. Recent meta-analyses point out that children exposed to opioids in the womb tend to have lower cognitive, language, and motor skills than those without such exposure [49,57].

However, these findings may be influenced by the small sample sizes, omission of other relevant factors, and confounding variables.

It is also essential to take into consideration the fact that pregnant women using opioids often face socioeconomic challenges, such as poor nutrition and unstable caregiving environments [49,50,57], which could partly explain developmental delays. Studies that adjust for these confounding factors generally find no significant differences in neurodevelopment due to prenatal opioid exposure alone [54,55,58].

**Figure 2:**
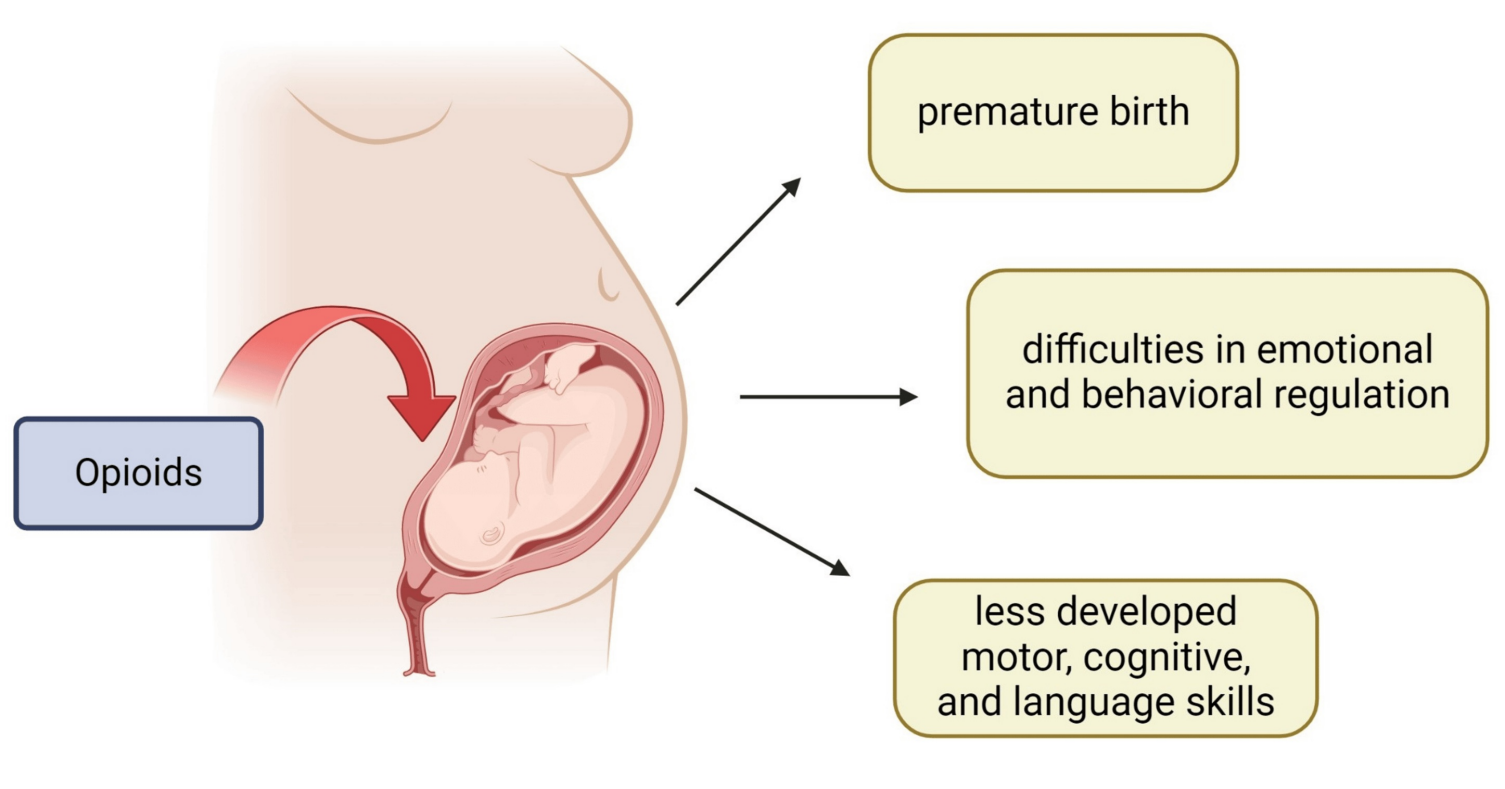
Impact of prenatal opioid exposure on fetal development and postnatal outcomes Source reference: [62] Created by Laura M. Marrelli using biorender.com

On the other hand, by age two, exposed children seem to struggle with different skills [59]. Children exposed to opioids during pregnancy often tend to have low scores in motor, cognitive [60], and language development and show more difficulties with self-regulation, emotional control, eating, feeding, and sensory processing compared to children who were not exposed [61,62]. These differences persist even after adjusting for maternal education and other substance use during pregnancy, pointing out that prenatal opioid exposure has a direct impact on child development. Additional postnatal family and parental factors accounted for 40-52% of the differences in developmental outcomes between exposed and unexposed children. Especially common are behavioral and emotional regulation issues, with exposed children being more likely to experience difficulties in multiple areas, including attention, emotional functioning, and sensory processing [62]. Figure 2 shows the impact of prenatal opioid exposure on fetal development.

Table 2 depicts the severity of the risk of opioid abuse and its effect on fetal development.

**Table 2:** Immediate and long-term risks of prenatal opioid exposure Source Reference: [59]

Risk type	Category	Findings
Immediate risk	Growth outcomes	Infants exposed to opioids are often born with lower average birth weights than those not exposed.
Immediate risk	Premature birth	Premature infants have had higher total opioid exposure during pregnancy.
Immediate risk	Neurodevelopmental scores	Most opioid-exposed infants score within the normal range on early neurodevelopmental tests, but perform worse in cognitive, motor, and behavioral assessments during early childhood.
Long-term risk	Cognitive, motor, and behavioral development	Children exposed to opioids in the womb tend to have lower cognitive, language, and motor skills than those without such exposure.
Long-term Risk	Language development	Exposed children tend to exhibit difficulties in language development, such as communication, expressive speech, and symbolic language.
Long-term Risk	Behavioral and emotional regulation	Children exposed to opioids exhibit more difficulties with self-regulation, emotional control, eating, feeding, and sensory processing.

Neonatal outcomes, including NAS and acute postnatal complications, including treatment

NAS is a withdrawal syndrome in newborns resulting from in-utero exposure to opioids, primarily due to maternal opioid use or treatment for opioid dependence during pregnancy [63]. This syndrome is characterized by symptoms, including central nervous system irritability, autonomic overactivity, and gastrointestinal dysfunction, and could appear in neonates within 24 to 72 hours after birth [63]. For NAS, the severity and duration can vary, often requiring medical intervention [63]. Treatment usually involves supportive care aimed at minimizing symptoms, with pharmacological therapy such as morphine or methadone being necessary in severe cases to stabilize the newborn and gradually wean them off opioids [63]. Drug concentrations from opioid use can be detected in neonates postnatally through Wharton’s jelly in the umbilical cord tissue and meconium [64]. Meconium testing is the gold standard that can detect higher concentrations of drugs that the fetus may have been exposed to during pregnancy [64]. Acute postnatal complications in infants with NAS include respiratory distress, feeding difficulties, and seizures, which are associated with the withdrawal process [63]. These complications are often exacerbated by prematurity and low birth weight, which are common in infants exposed to opioids in utero [63]. The management of these complications requires a multidisciplinary approach, including respiratory support, nutritional assistance, and, in some cases, anticonvulsant therapy, highlighting the importance of early and comprehensive care for these infants [63].

**Figure 3:**
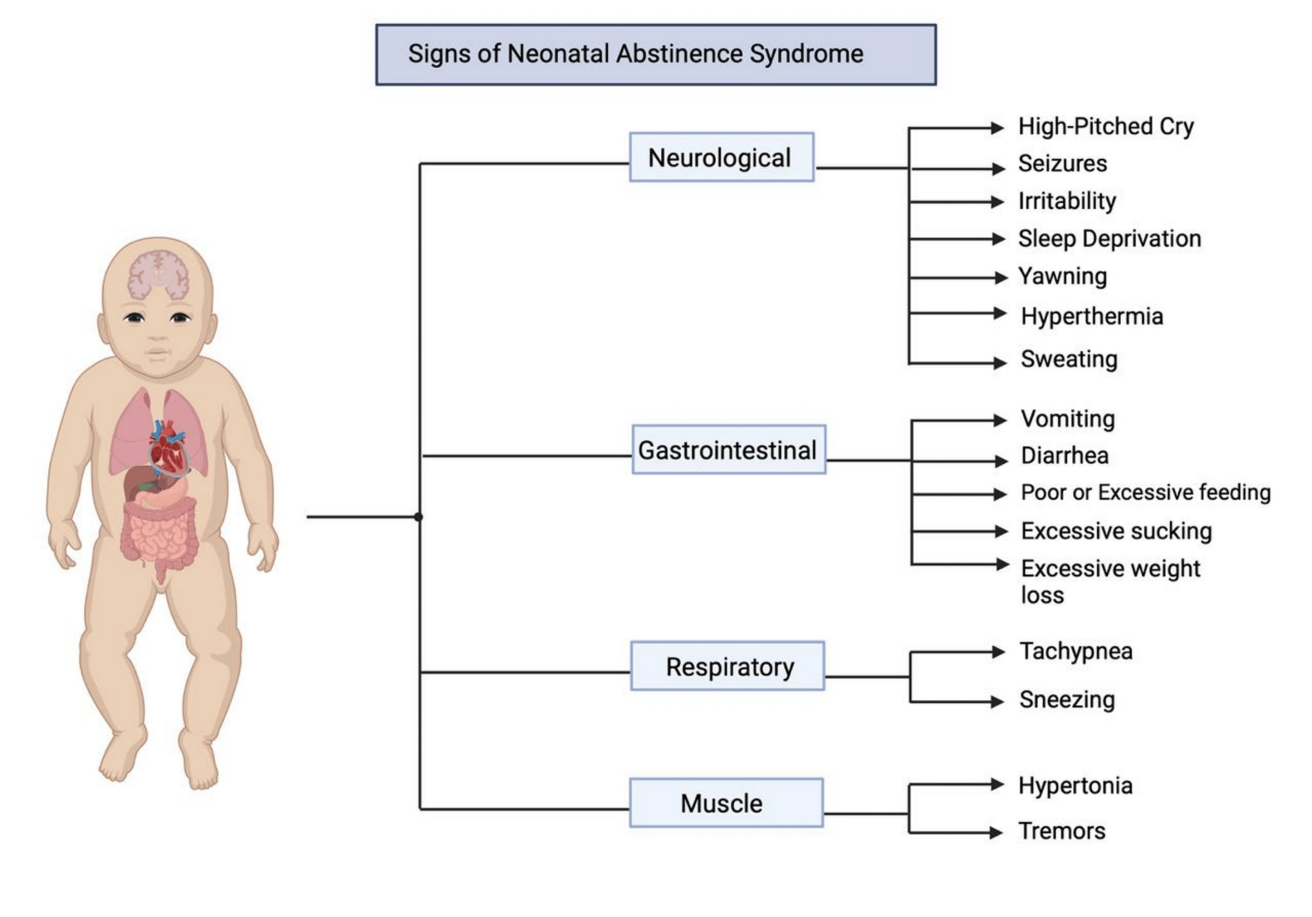
Signs of neonatal abstinence syndrome in an infant. Created by Neisha Ghanie using biorender.com

Pharmacological treatment for NAS typically involves the administration of opioids such as morphine or methadone to alleviate withdrawal symptoms [63]. The dosage is carefully titrated based on the severity of symptoms, as assessed by scoring systems like the Finnegan Neonatal Abstinence Severity Score [63]. Gradual tapering of the medication is then implemented to minimize withdrawal effects as the infant’s dependence on opioids decreases [63]. Non-pharmacological interventions, including rooming-in with the mother, skin-to-skin contact, and breastfeeding, are also crucial in managing NAS, as they can reduce the severity of symptoms and the need for pharmacological treatment [63]. As opioid agonist therapy is the standard of care for OUD in pregnancy, it may also contribute to the NAS diagnosis postnatally; however, it contributes to shorter hospital stays and treatment for NAS [65]. Long-term outcomes for infants with NAS are influenced by several factors, including the severity of the syndrome, the type and duration of treatment, and additional risk factors such as exposure to multiple substances or poor postnatal care [63]. Various research depicts that infants with NAS are at risk for developmental delays, particularly in motor skills and cognitive function, which may persist into childhood [63]. Early intervention programs and continuous monitoring are essential to address these potential developmental issues and support the child’s growth and development [63]. Figure 3 depicts the signs of NAS in an infant.

Maternal opioid use treatment approach

Current WHO guidelines support opioid maintenance treatment (OMT) for MOUD during pregnancy, using methadone or buprenorphine [66]. Methadone has been recommended since the 1970s to stabilize pregnant women with OUD because the fetus is shielded from the adverse effects of repeated withdrawals that are common in the setting of opioid abuse by a steady concentration of opioids in the mother's bloodstream. It has been demonstrated to maintain patients in treatment better and reduce heroin use in OUD patients when compared to no opioid replacement therapy. Methadone has the added advantages of boosting prenatal visits, lowering HIV infection rates, and lowering preeclampsia rates in pregnant women who are illicit opiate users [22]. However, methadone use may potentially result in drug-drug interactions with other medications. It can build up in slow metabolizers, putting patients at risk for respiratory depression and long QT syndrome (torsades de pointes) at high systemic levels. When compared to infants whose mothers continue to use illicit opiates such as heroin, the fetus benefits from lower odds of preterm delivery, fetal loss, and low-birth-weight infants. Women in pregnancy have two options: continue an already established dose of methadone or start methadone during the pregnancy [22].

Since 2002, buprenorphine has been authorized for use in medically supervised withdrawal and maintenance therapy [22]. Maternal and fetal outcomes were not substantially different from those of women treated with buprenorphine alone. According to small retrospective research including 10 pregnant women who received both buprenorphine and naloxone, days of treatment for NOWS, length of hospital stay, and admission rate to the neonatal intensive care unit were similar in both groups. In another retrospective study, 62 mother-infant dyads were included; 31 of them received methadone treatment, and 31 of them received buprenorphine and naloxone treatment. The group receiving buprenorphine and naloxone treatment showed significantly lower rates of NOWS, shorter hospital stays, and lower peak NOWS scores in the affected infants when compared to the infants who received methadone treatment [22]. A recent Cochrane review highlighted that the total body of evidence is modest and that while methadone looked to be more likely to keep individuals in treatment, buprenorphine appeared to have better neonatal withdrawal outcomes [22]. The Maternal Opioid Treatment: Human Experimental Research Study (MOTHER) trial looked into opioid agonist pharmacotherapy using buprenorphine or methadone. Research suggests that newborns of buprenorphine-maintained mothers receive a lower maternal dosage compared to methadone. This could explain why buprenorphine-exposed newborns experience fewer withdrawal symptoms [67]. 

Naltrexone is a competitive agonist at opiate receptors that inhibits the euphoric effects of opioids, lowering the perceived benefits of illicit opioid use. There is limited data on its use during pregnancy. However, the safety profile appears to be positive at present. Naltrexone is not linked to NOWS. The fact that complete opioid detoxification is required prior to initiating naltrexone limits its use during pregnancy. Compared to patients who go through detoxification, pregnant patients with OUD on MAT (medication-assisted treatment) show higher rates of engagement with treatment and abstinence from illicit drugs. The Substance Abuse and Mental Health Services Administration (SAMHSA) and the American College of Obstetricians and Gynecologists (ACOG) currently recommend MAT as the initial treatment option for pregnant women who are actively using opioids. When they find out they are pregnant, many women receiving maintenance opioid replacement treatment want to undergo detoxification. However, in earlier research, rapid opioid withdrawal was linked to several problems, including increased rates of miscarriage, fetal discomfort caused by withdrawal symptoms, and preterm labor.

Nevertheless, the majority of research on detoxification during pregnancy is underpowered and does not include control groups, and a sizable percentage of patients are lost to follow-up [22]. While the ACOG supports opioid agonist medication as the treatment for OUD in pregnancy, SAMHSA also suggests psychosocial support, which should, at the very least, involve a needs assessment and a referral to more comprehensive psychosocial treatment services. Regretfully, there is a paucity of consistent research on the advantages of behavioral therapies, either in place of or in addition to MAT, for expectant mothers [22]. A state's reaction to prenatal substance use can be determined by a variety of policy initiatives, which may either encourage or discourage timely admission to treatment for OUD [68]. The low MOUD receipt rate among individuals referred by criminal or legal bodies suggests that this referral pathway has to be strengthened. These results may contribute to a better knowledge of the treatment referral sources that can serve as efficient routes to MOUD, hence fostering improved outcomes for women and families dealing with substance abuse [68]. 

The adoption of universal screening techniques for drug use disorders early in pregnancy is supported by these findings; nevertheless, further interventions are required to guarantee that patients with OUD receive evidence-based and culturally sensitive therapy. Reducing this disparity in MOUD receipt is a crucial first step toward achieving equity in health consequences for pregnant and postpartum women with OUD, given the health advantages of MOUD during pregnancy and the postpartum period [24].

Pain management in opioid-dependent pregnant women

Pain management in opioid-dependent pregnant women includes continual OMT throughout the pregnancy and postpartum period, adequate management of acute pain, and contraindication of opioid agonist-antagonists for pain management. The prevalence of opioid use among pregnant women is increasing concurrently. The percentage of pregnant women pursuing treatment for addiction related to prescription opioids has risen from 2% to 28%.

Opioid drug tolerance refers to the long-term use of opioids, causing an increased dosage requirement to achieve the original effect of the drug to control pain in patients receiving OMT adequately. Cross-tolerance refers to tolerance for one drug leading to tolerance for another. The opioid-dependent patient's tolerance for methadone created a cross-tolerance of morphine.

Studies were conducted with buprenorphine, a partial mu opioid agonist, and kappa opioid antagonist [69]. The high affinity of buprenorphine for μ-receptors allows for minimal displacement of the drug from the receptor by other opioids. In the treatment of addiction, this high affinity is protective against the risk of misuse and overdose. In treating pain, however, the strong bond between buprenorphine and the μ-receptor may lead to inadequate analgesia despite increased dosing of opioids. As a result, cessation of buprenorphine is recommended at least two days prior to procedures. However, it is recommended not to discontinue buprenorphine to manage acute pain in opioid-dependent pregnant women because of the difficulty in restarting maintenance therapy [70]. There is some evidence that acute pain in women receiving BMT may be managed adequately in pregnancy with short-acting opioids and, in the postpartum period, with short-acting opioids and nonsteroidal anti-inflammatory drugs (NSAIDs). Providing the required higher opioid dosage to opioid-dependent patients is often limited by a concern for dose-dependent respiratory and central nervous system effects, such as respiratory depression or decreased consciousness [71]. There is some evidence that opioid-maintained pregnant women may suffer less significant dose-related respiratory and central nervous system effects. In opioid-dependent women, NAS is caused by withdrawal from in-utero drug exposure [71,72]. The occurrence of NAS and duration of treatment of NAS did not differ between methadone- and buprenorphine-exposed newborns [73]. 

Pain management in pregnant women receiving OMT: the clinical aspect

Antepartum

This section addresses five critical aspects of antepartum pain management: pain prevention, common complaints of pregnancy, acute pain, chronic pain, and the pain management plan. It is always advisable to continue OMT in opioid-dependent pregnant women instead of discontinuing them. Pain prevention strategies include mental healthcare needs, such as evaluating sleep hygiene and developing a plan to improve sleep quality and progress on smoking cessation.

Pain Management Strategies

It is recommended to change the routine OMT dosage according to their opioid tolerances in opioid-dependent pregnant women. Given the adverse effects of the drugs (opioids), it is advisable to enhance the nonpharmacologic and nonopioid ways of pain relief. A short course of opioid agonist medication like morphine et al./acetaminophen hydrocodone or oxycodone is acceptable to treat acute pain. If this medication does not control the pain, they can be referred to a pain management specialist [74]. 

During chronic pain in pregnancy, a multidisciplinary approach is preferred.

Intrapartum

Chronic use of opioids causes tolerance and hyperalgesia. Epidural and spinal analgesia can be used safely in these patients. In the case of cesarean delivery, general anesthesia is preferred. Nalbuphine (Nubain) and butorphanol (Stadol) should be avoided in opioid-dependent patients, as they will precipitate withdrawal.

For pain of short duration, patients may discontinue their buprenorphine prior to labor and restart again later, and their pain can be managed with short-acting opioids as needed [75]. 

If buprenorphine is continued up until labor, neuraxial anesthesia is preferred for labor. Higher doses of short-acting opioids may be required, and respiratory depression should be monitored since it could be a side effect. Spinal or epidural morphine can also be given postoperatively for pain management. 

Postpartum

A retrospective medical record review conducted in Australia showed that 74% of opioid-dependent women undergoing cesarean delivery had inadequate postoperative pain control. They found that women receiving OMT experienced increased postpartum pain when compared to opioid-naive women, which indicates worse pain management. The data suggest that significantly more opioids are dispensed to the opioid-naive group post-cesarean delivery and reticence on the part of providers to dispense opioids to women receiving OMT.

Postpartum pain in buprenorphine-maintained women was adequately treated with opioids and NSAIDs. Nicotine replacement and other adjuvant methods (e.g., heating pads, ice packs, sitz baths, and ambulation) can also improve women's comfort [76]. 

An essential part of pain management during discharge is continuing as per discharge advice and maintaining follow-up appointments. This treatment should be provided in a caring and non-punitive atmosphere, and public health authorities, legislators, and doctors all have a role to play in making this happen [77].

Six categories of pertinent policies are looked at in Table 3 [77].

**Table 3:** Elements of state policy analyzed to address maternal opioid use disorder (OUD) SUD: substance use disorder

Elements of state policy
Classify substance use during pregnancy as child abuse or neglect, criminalize it or consider it grounds for civil commitment
Testing of newborns suspected of prenatal substance exposure or pregnant women suspected of substance use
Reporting suspected prenatal substance use to local human services and health department officials
Create/fund targeted programs for pregnant and postpartum women with substance use disorders
Prioritize pregnant women's access to SUD treatment programs
Forbid discrimination against pregnant women in publicly funded SUD treatment programs

There are positive instances of wise policy at the state and federal levels [78]. The significance of recent federal legislation cannot be overstated. These laws have addressed gaps in the continuum of care for pregnant and postpartum women and have strengthened plans of safe care for infants with prenatal substance exposure, taking a much-needed public health approach to this issue [78].

The special issue was sponsored by the Association of Maternal & Child Health Programs (AMCHP) and the Association of State and Territorial Health Officials (ASTHO), with funding provided by the US Maternal and Child Health Bureau Health Resources and Services Administration (HRSA-MCHB). Through the PRISM (Promoting Innovation in State and Territorial MCH Policymaking) project, AMCHP and ASTHO have worked with states to advance public health policies to address perinatal SUD [79].

Essential takeaways from a selection of position papers, policy declarations, panel reports, and guidance documents about substance use during pregnancy are depicted in Table 4.

**Table 4:** Key insights regarding the management of substance use disorder (SUD) during pregnancy, derived from position papers, policy statements, panel findings, and advisory documents

Organization	Crucial elements
American Academy of Family Physicians (AAFP) [80]	“The AAFP refrains from imprisonment or other criminal sanctions of pregnant women only for substance use during pregnancy, but encourages facilitated access to drug and alcohol rehabilitation programs for them.”
Centers for Disease Control and Prevention (CDC) [81]	“Public health interventions to prevent and treat opioid dependence at the time of pregnancy are important to diminish the incidence of NAS and its related health care burden.”
Substance Abuse and Mental Health Services Administration (SAMHSA) [82]	"women who have substance use disorders often fear prosecution and incarceration if they seek treatment during pregnancy.”
World Health Organization (WHO) [83]	“Interventions should be provided to pregnant and breastfeeding women in ways that prevent stigmatization, discrimination, criminalization, and marginalization of women seeking treatment to benefit themselves and their infants. Prevention and treatment should promote and facilitate family, community and social support as well as social inclusion by fostering strong links with available childcare, economic supports, education, housing, and relevant services.”
Office of Women’s Health (OWH) [84]	“Involvement with the child welfare system plays a critical role in a woman’s decision to seek care, because admitting to a substance use disorder may lead to involvement with the criminal justice system and potential loss of custody. The 2011 National Drug Control Strategy has acknowledged the importance of women not having to choose between pursuing treatment and caring for their children.”

Preliminary program assessments and detection and therapy of opioid use during gestation are the main focuses of this supplement, mainly because of jurisdictional, federal, and national financial and strategic concerns. Nevertheless, the writers acknowledge the effects of tobacco, alcohol, and cannabis on the health of expectant mothers and their offspring. Subsequent studies and financial support have to concentrate on polysubstance use throughout pregnancy and the postpartum phase and find ways to offer these people complete care and therapy [85].

Future direction and research gaps

OUD continues to be highly stigmatized, and the impact of the social stigmata of OUD cannot be disregarded, as they do not exist in isolation but instead are interconnected. It is further compounded by marginalization associated with race, gender, ethnicity, sexual orientation, age, and socioeconomic status. The stigmatization of OUD is multifaceted, with individuals of injection opiates being confronted with various types of stigmata encompassing societal, public, and their internalization, leading to self-stigma.

This ultimately results in delays in obtaining treatment and an increased rate of premature treatment withdrawal, jeopardizing recovery and reintegration with mainstream society. To mitigate this stigmatization, organizational leaders and policymakers are in a key position to promote and reinforce destigmatizing language surrounding OUD.

More large-scale stigmatization campaigns are required since they have the potential to transform societal and cultural perceptions of OUD, leading to more significant results on the continuum of care. Furthermore, additional research is required to evaluate the effectiveness of these interventions to reduce stigma [82].

Examining modifications in placental signaling in the aftermath of long-term opioid usage is another potentially productive line of inquiry [11]. Assessment of different serum concentrations of metabolites derived from placentas, such as endorphins, bone morphogenetic proteins, and microRNAs, may disclose novel targets for therapy that are targeted specifically at reducing the risk of neurodevelopmental abnormalities in infants caused by prolonged exposure to opioids during pregnancy. Although placental-derived metabolites are not now the subject of approved placental therapeutics, interest in this area is growing [11]. In addition, it has generally been difficult, both logistically and ethically, to convert preclinical research of such placental therapies into clinical trials [11]. The placental structure of different mammals, such as human beings, pigs, sheep, and mice, varies greatly; it is uncertain if preclinical animal studies in exploratory placental treatments are valid [83,84]. However, because exploratory placental treatments include two members of vulnerable patient populations-the pregnant mom and the fetus-they also come with a higher risk of adverse effects [11]. The growing need for placental/fetal therapies in the United States justifies prospective preclinical and clinical research in the rising incidences of NOWS and NAS secondary to the opioid epidemic, even though these barriers are still significant and will undoubtedly lead to limitations in future studies [11].

With the added benefit of finding novel genetic variants and biomarkers that correlate with specific phenotypes, candidate gene studies and genome-wide association (GWA) analysis can be used to explore genetic influences on drug addiction and dependence [85]. A solid biological basis for the emergence of addiction may be provided by this understanding and integration of genetic and epigenetic data [85]. Sorting people with varying propensities for addiction into physiologically based groups would also benefit immensely from a focus on biomarkers with predictive solid and explanatory value [86]. These biomarkers are changes in biology or chemistry that can be reliably rated and evaluated as a sign of pathology, pharmaceutical response, or disease diagnosis [87].

A lot more study needs to be done to characterize the combination of genetic and environmental variables that contribute to a person's susceptibility to addiction if they were exposed to opioids during pregnancy [85].

It should be the top priority for everyone involved in clinical care and research to give expectant mothers with OUD or other health conditions requiring opioid drugs as many chances as possible to promote the development of their fetus while navigating therapy [11]. It also raises the subject of whether the existing OMT is lacking in any way or if the higher rates are a result of people's intentions or are connected to stress and worry [88]. Despite OMT, more research must be done to determine the source and consequence of this rise in fentanyl use [88]. Furthermore, the factors that contribute to racial and ethnic differences in medicine for OUD (MOUD) use during pregnancy are mainly unknown. The healthcare system's systemic racism, which preserves the privilege of White people, is represented by additional structural barriers that nonwhite groups experience when seeking healthcare. These include a paucity of healthcare providers servicing varied communities. These cultural preconceptions result in a lack of or poor quality care from providers and job and housing restrictions that contribute to socioeconomic disparities [24]. The implementation of universal screening for substance use during pregnancy is imperative; nevertheless, additional interventions are required to guarantee equal access to culturally competent, evidence-based care. For all pregnant and postpartum women with OUD, addressing these inequities is essential to promoting fairness and better health outcomes [24].

## Conclusions

The impact and challenges of OUD have been an emergent area of concern over the years, as OUD has increased significantly and is among the many health crises that healthcare professionals, policymakers, and legislators have to confront.

With the increased prevalence of maternal opioid use, both prescription opiates and heroin, the psychological impact of opioid dependence is concerning as it poses many challenges in the public health sector.

As maternal OUD is often compounded by co-exposure and abuse of other non-opioid substances, there are numerous deleterious effects and clinical implications for the fetus, infant, and childhood neurodevelopment.

Although there has been a decrease in punitive approaches in the US, revisions and improvement are still warranted in the criminalization of maternal SUD by some states, which can be prohibitive in this marginalized group seeking assistive treatment and prenatal care. As such, a multidisciplinary approach is required to tackle the surge of opioid use and the numerous maternal, fetal, neonatal, and long-term childhood sequelae.
